# Deep learning-based automatic sella turcica segmentation and morphology measurement in X-ray images

**DOI:** 10.1186/s12880-023-00998-4

**Published:** 2023-03-25

**Authors:** Qi Feng, Shu Liu, Ju-xiang Peng, Ting Yan, Hong Zhu, Zhi-jun Zheng, Hong-chao Feng

**Affiliations:** 1 College of Medicine, Guizhou University, Guiyang, 550025 China; 2 Department of Orthodontics, Guiyang Hospital of Stomatology, Guiyang, 550002 China; 3 Department of Radiology, Guiyang Hospital of Stomatology, Guiyang, 550002 China; 4 Department of Medical Information, Guiyang Hospital of Stomatology, Guiyang, 550002 China; 5 Department of Oral and Maxillofacial Surgery, Guiyang Hospital of Stomatology, Guiyang, 550002 China

**Keywords:** X-ray images, Sella turcica, Deep learning, OpenCV, Automatic segmentation, Automatic measurement

## Abstract

**Background:**

Although the morphological changes of sella turcica have been drawing increasing attention, the acquirement of linear parameters of sella turcica relies on manual measurement. Manual measurement is laborious, time-consuming, and may introduce subjective bias. This paper aims to develop and evaluate a deep learning-based model for automatic segmentation and measurement of sella turcica in cephalometric radiographs.

**Methods:**

1129 images were used to develop a deep learning-based segmentation network for automatic sella turcica segmentation. Besides, 50 images were used to test the generalization ability of the model. The performance of the segmented network was evaluated by the dice coefficient. Images in the test datasets were segmented by the trained segmentation network, and the segmentation results were saved in binary images. Then the extremum points and corner points were detected by calling the function in the OpenCV library to obtain the coordinates of the four landmarks of the sella turcica. Finally, the length, diameter, and depth of the sella turcica can be obtained by calculating the distance between the two points and the distance from the point to the straight line. Meanwhile, images were measured manually using *Digimizer*. Intraclass correlation coefficients (ICCs) and Bland–Altman plots were used to analyze the consistency between automatic and manual measurements to evaluate the reliability of the proposed methodology.

**Results:**

The dice coefficient of the segmentation network is 92.84%. For the measurement of sella turcica, there is excellent agreement between the automatic measurement and the manual measurement. In Test1, the ICCs of length, diameter and depth are 0.954, 0.953, and 0.912, respectively. In Test2, ICCs of length, diameter and depth are 0.906, 0.921, and 0.915, respectively. In addition, Bland–Altman plots showed the excellent reliability of the automated measurement method, with the majority measurements differences falling within ± 1.96 SDs intervals around the mean difference and no bias was apparent.

**Conclusions:**

Our experimental results indicated that the proposed methodology could complete the automatic segmentation of the sella turcica efficiently, and reliably predict the length, diameter, and depth of the sella turcica. Moreover, the proposed method has generalization ability according to its excellent performance on Test2.

## Background

The sella turcica (ST) is a saddle-shaped structure in the middle cranial fossa on the intracranial surface of the sphenoid bone, which can be readily recognized in lateral cephalometric radiographs. ST is routinely used to evaluate cranial morphology and determine the relationship of the maxilla and mandible with each other or the cranial base [1]. The anterior border of ST is the tuberculum sellae and the posterior border is the dorsum sellae. In orthodontics, the sella point, which is located at the center of ST, is one of the most widely applied cranial landmarks for cephalometric analysis [2]. During embryological development, ST area is the key site for cranial and maxillofacial development. The neural crest cells migrate from ST to the fronto, nasal and maxillary developmental fields, and are involved in the formation and development of the anterior part of the pituitary gland, ST, and teeth. This makes morphometrics of ST a good source of additional diagnostic information related to pituitary pathology, dental abnormalities or various syndromes that affect the craniofacial region [3].

In the field of stomatology, the study of the morphological changes of ST have been drawing increasing attention from stomatologists. Several studies conducted on the association between variation in ST morphology and skeletal malocclusions [4–6]. Some authors have performed the morphometric analysis of ST in populations of different races, ages, and genders, to identify possible race, age, and gender correlation with ST size [2, 7]. Other studies have explored whether genetic syndromes involving the craniofacial complex and the presence of dental abnormalities are associated with abnormal radiographic ST morphology [8–10]. All the studies mentioned above include the same essential step: measuring the morphology of ST.

Linear measurements such as length, depth, and diameter were the most common parameters to assess the morphology of ST. This morphological method to measure the linear size of ST was first proposed by Silverman [11] and is still used in current studies. Observers usually manually measure these linear parameters using digital calipers or measurement software. However, manual measurement is a time-consuming and laborious task. In addition, both inter-observer and intra-observer variabilities are prone to exist during the measurement process. These disadvantages of manual measurement will hinder the study of ST morphology, so it is necessary to provide a convenient automatic measurement tool.

Deep learning, a powerful emerging branch of artificial intelligence technology, has made many achievements in medical image processing in recent years. Qin Ye et al. developed a deep learning-based system for automatic patellar height measurements using knee radiographs [12]. Woo et al. [13] developed a deep learning algorithm for semi-automated unidirectional CT measurement of lung lesions. Ahmad et al. [14] proposed a very lightweight convolutional neural network (CNN) to extract the liver region from CT scan images. And some other studies are based on deep learning algorithms, such as automatic segmentation and measurement of vertebral [15–17], automatic measurement of eyelid morphology [18, 19], and automatic measurement of hip joints [20], etc. This study aims to develop and evaluate a deep learning-based model for automatic segmentation and measurement of ST in cephalometric radiographs.

## Methods

### Development environment

In our experiments, we use NVIDIA RTX 3090 graphics processing units to help accelerate deep learning training. The deep learning development environment was done through Python 3.7.11 and Pytorch 1.10.2 framework in Windows 10 operating system.

### Datasets

The X-ray images we use were obtained from the PACS in the Imaging Department of Guiyang Hospital of Stomatology. In the process of data collection, the morphological characteristics of ST on each image were carefully observed. Images that clearly showed the morphological characteristics of ST were included in the study, while those of poor quality were excluded. After the screening, cephalometric radiographs of 1129 patients were included in the study, constituting our experimental datasets. The 1129 X-ray images were exported from the PACS system and saved in JPG format. The size of each image is 2884 × 2304 pixels, and each pixel’s size is 0.1 × 0.1 mm. 110 images were randomly selected from 1129 images as a test dataset, named Test1.The remaining 1019 X-ray images were randomly divided into two sets: 918 images were used as the training dataset to train the deep learning network for automatic ST segmentation, and 101 images were used as the verification dataset.

In addition, from the 400 X-ray images in the 2015 ISBI public dataset, 50 images were selected that could clearly show the morphology of the ST. The 50 images constitute dataset Test2 as the second test dataset to test the generalization ability of the proposed method. The original format of the 50 images was BMP, which was converted into JPG format in this study. The size of the images was 1935 × 2400 pixels, and each pixel's size is 0.1 × 0.1 mm.

Automated morphological measurements for the ST were performed on Test1’s 110 images and Test2’s 50 images. The images in the test dataset are input the trained deep learning network, the region of the ST in each image is automatically segmented, and an algorithm is designed to achieve automatic morphological measurements of the ST on the segmentation results.

### Definitions of measures

The method of measuring the linear dimensions of ST was first proposed by Silverman [11]. There are four landmarks on the contour of the ST: 1. The tuberculum sella (TS), which is the anterior point of the contour of the ST; 2. The dorsum sella (DS), which is the highest point of posterior wall of the ST; 3. The base of the pituitary fossa (BPF), which is the deepest point on the floor of the pituitary fossa; 4. The anteroposterior diameter (ADP), which is the furthest point on the posterior inner wall of the fossa [2]. Based on these four landmarks, Silverman defined three linear parameters (Length, Diameter and Depth) to describe the morphology of the ST.

**Fig. 1:**
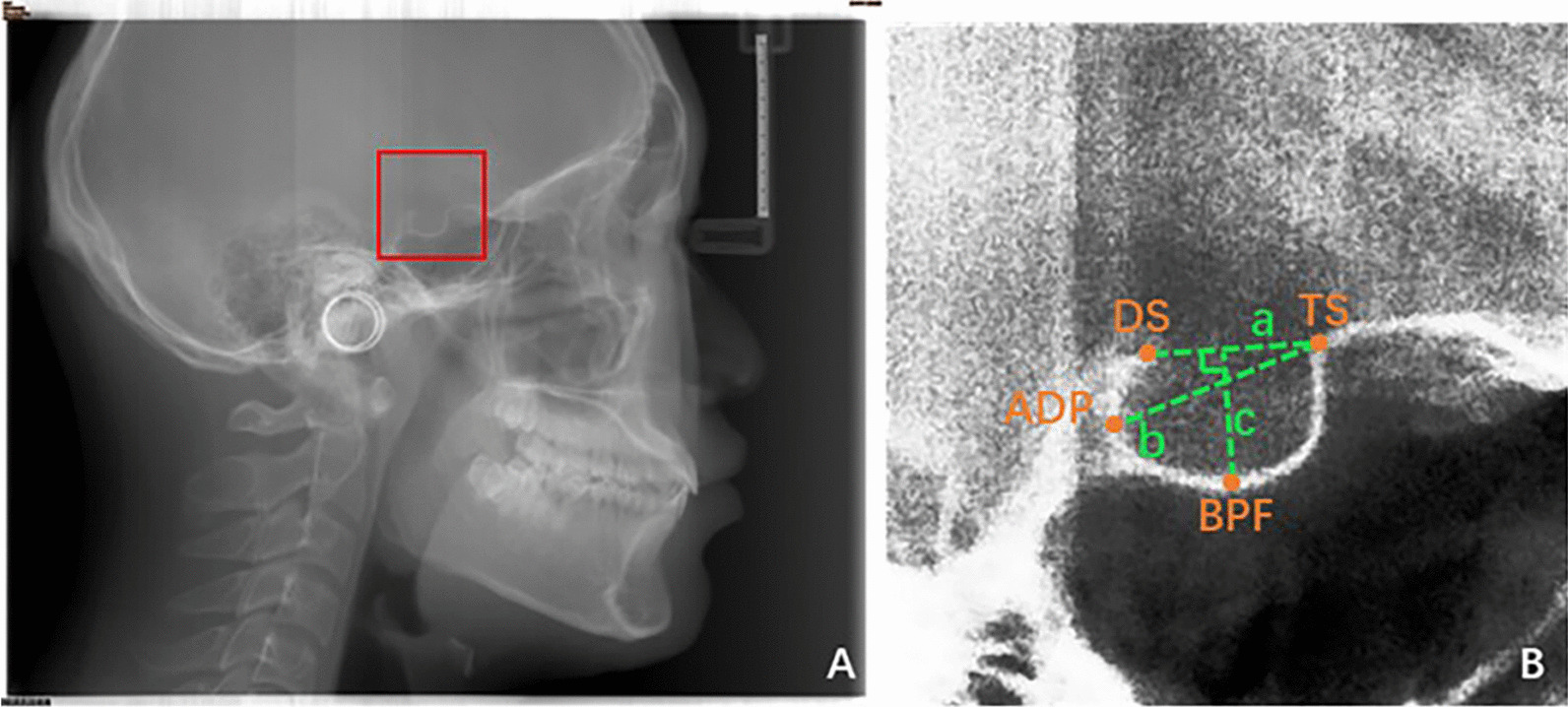
Dimensions of Sella Turcica. **A** An original X-ray image. The structure in the red box is the ST. **B** The region of the ST. TS, DS, ADP, and BPF were the four landmarks of ST, line a is Length, line b is Diameter, and line c is Depth

The length of the ST is the distance from TS to DS. The anteroposterior diameter of the ST is the distance from TS to ADP. The depth of the ST is the vertical distance from BPF to a straight line formed by TS and DS connections. The positions of the four landmarks of the ST in the image and the specific definitions of the three linear parameters as shown in Fig. 1.

### Preprocessing

**Fig. 2:**
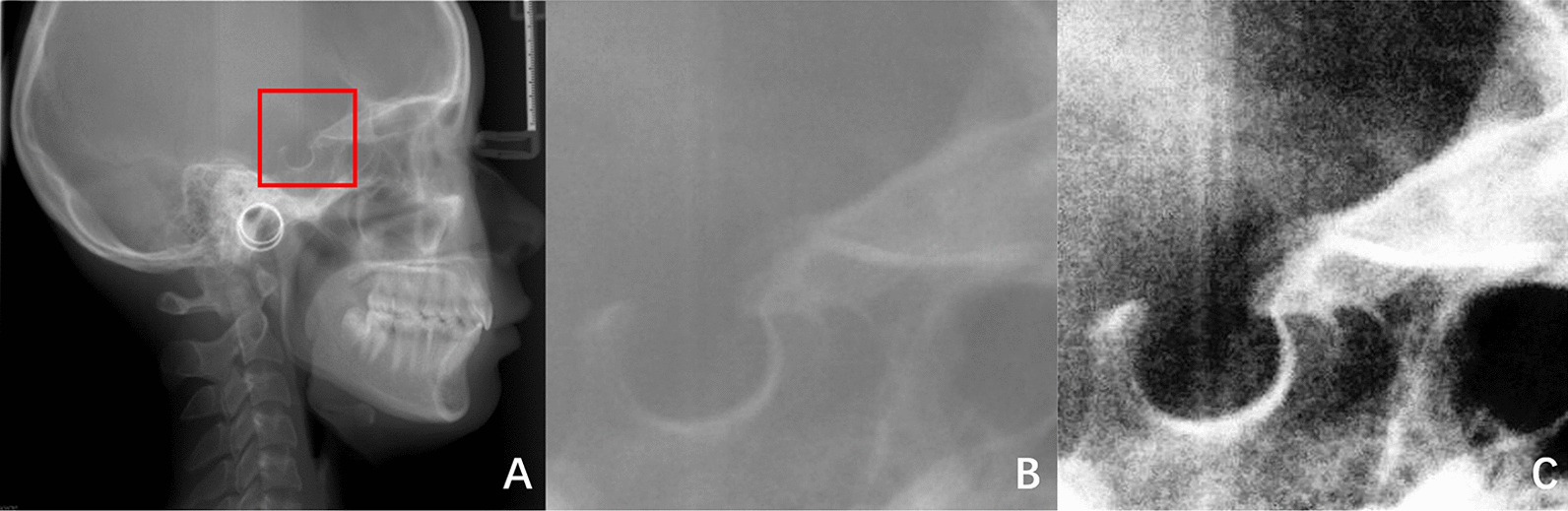
An example of image preprocessing. **A** An original X-ray image. **B** Result of extracting ROI. **C** Result of histogram equalization and median filtering

Preprocessing is a vital part of image segmentation, and many studies add a preprocessing phase before training deep learning models [21–23]. In this study, all images are preprocessed before training the network for automatic ST segmentation. There are three main preprocessing operations. The first preprocessing operation is to extract the region of interest (ROI). The size of the original image was 2884 × 2340 pixels, but the ST as the target for segmentation only occupied a small area of the whole image. Therefore, the region containing the ST was extracted from the image, and irrelevant background was removed. By calling the function in OpenCV library, the ROI is extracted based on pixel coordinate parameters. The pixel coordinate parameters are “image [*y*_0_:*y*_1_, *x*_0_, *x*_1_]”, where (*x*_0_, *y*_0_) is the top-left coordinate of ROI, and(*x*_1_, *y*_1_) is the bottom-right coordinate of ROI. There are two reasons why pixel coordinates can be used to extract ROI. One reason is that the cephalometric radiographs taken with the same device are generally of the same size. Another reason is that although the morphology of the ST varies from person to person, the relative position of the ST in the cephalometric radiographs does not change much. Therefore, as long as the appropriate pixel coordinate parameters are set, the ROI can be easily and quickly extracted by traversing all images in the dataset using OpenCV. For our dataset, we set the pixel coordinate parameters to [500:900, 1200:1600], traversed and processed all the images in our dataset by calling functions in OpenCV. For Test2 selected in the public dataset, we changed the pixel coordinate parameters to [800:1200, 600:1000]. The ROI of all images was quickly extracted and accurately included the ST. The size of ROI is 400 × 400 pixels. The second preprocessing operation is histogram equalization. The purpose of this operation is to enhance the contrast of the image and make the morphological structure of the ST clearer in the image. The third preprocessing operation is median filtering, which aims to remove noise from the image. The median filtering can not only remove some isolated noise points in the image, but also maintain the image details. In our experiment, the “ksize” of median filtering was set to 5. After preprocessing, all images are 400 × 400 pixels in size. Figure 2 shows an example of image preprocessing.

### Annotations

1. Manual segmentation of ST

**Fig. 3:**
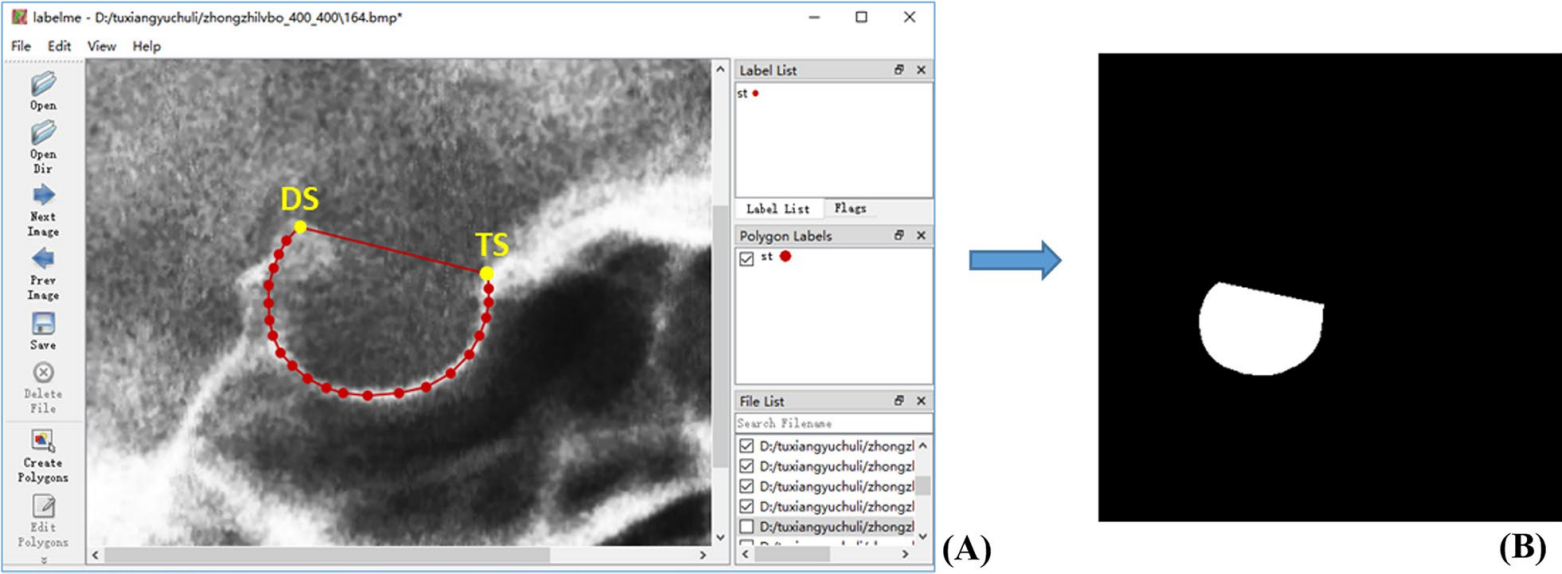
An example of image annotation. **A** ST was manually segmented and annotated using *Labelme.* **B** Corresponding mask

The main researcher used *Labelme* (version 5.0.1), a common program for labeling images, to manually segment the ST. After annotation, a corresponding mask will be generated for each image, as shown in Fig. 3. In the X-ray images, ST appears as a nest bounded by a circular white line. The ST in the image is manually segmented along the white line. The specific rules of annotation are as follows: take TS as the starting point and DS as the ending point, draw along the inner edge of the white line, and then connect DS and TS. Finally, the closed region formed is the segmented ground truth. All images were annotated by the same researcher, and an experienced orthodontist was invited to review the annotation results to ensure the reliability of ground truth. Disputed images are discussed and re-labeled.

2. Manual measurement of linear parameters

*Digimizer* is an easy-to-use image analysis software package that allows precise manual measurements. X-ray images in Test1 and Test2 were manually measured using *Digimizer* (version 5.4.4), and the Length, Diameter, and Depth of ST of each image were obtained. The 110 images in Test1 and 50 images in Test2 were measured three times by the same observer, with an interval of two weeks between each measurement. The average value of the three measurements was taken as the final manual measurement result, which was used as the “gold standard” to check the reliability of the automated measurement method proposed in this study.

### Training U-net for automatic segmentation of ST

**Fig.4:**
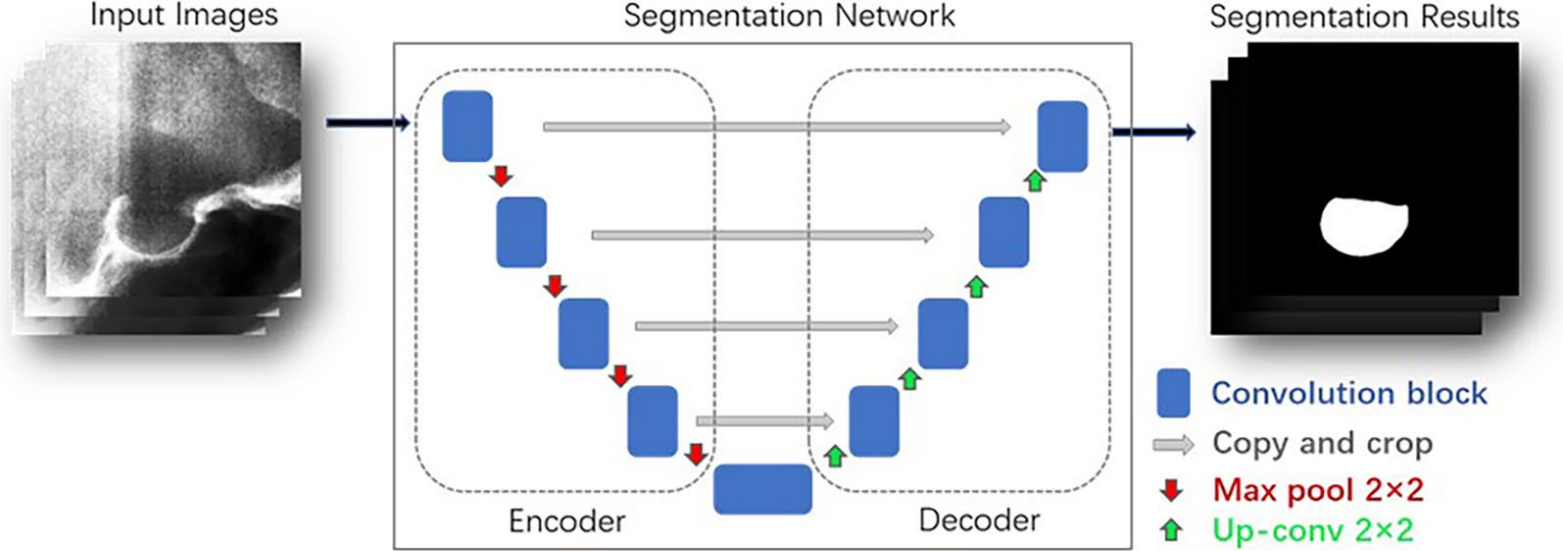
The U-net model for automatic segmentation of ST

U-net is a Fully Convolution Network (FCN) mainly composed of convolutional layers, pooling layers, transposed convolutional layers, skip connections, and nonlinear activation function ReLU. U-net is widely used to medical image segmentation, and its U-shaped structure has the advantages of combining context information and fast training speed [24]. In this study, 1019 images and their corresponding masks were input into the U-net model for training and validation. The input images have been preprocessed and the size of each image is 400 × 400. The BCEWithLogitsLoss loss function was adopted and the RMSprop optimizer was used in all training processes. The learning rate is set to be 0.00001 with call back function ReduceLROnPlateau, batch size of 8, and epochs of 50. The trained U-net model can realize automatic segmentation of ST, as shown in Fig. 4.

Dice coefficient, a typical evaluating indicator to verify the automated image segmentation model [15], was used to evaluate the segmentation performance in this study. The dice coefficient is defined as follows:

1 $$Dice = \frac{{2\left| {A \cap B} \right|}}{{\left| A \right|{ + }\left| B \right|}}$$

Here A is a set representing the ground truth and B represents the computed segmentation [25]. The model with the best segmentation performance on the verification dataset was saved as the best model. The images in Test1 and Test2 are preprocessed before being input into the segmentation network, and the size of each image is 400 × 400. The ST of all images in Test1 and Test2 was automatically segmented by the best model, and the binary segmentation results are saved.

### Postprocessing

**Fig.5:**
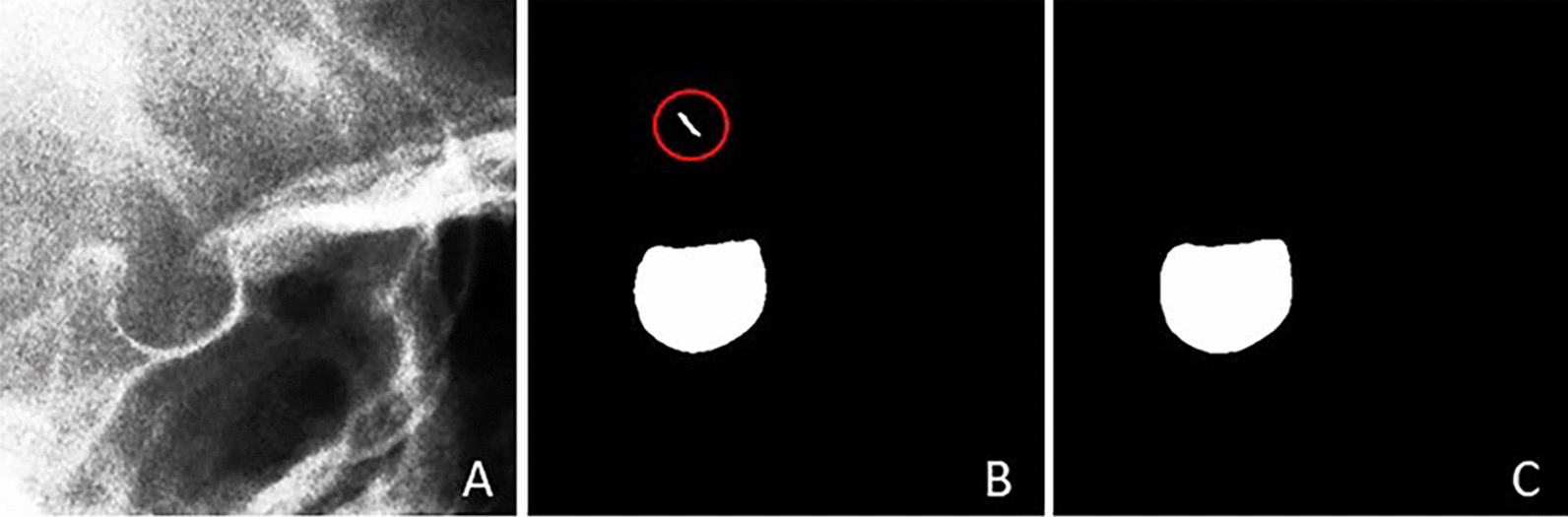
Example of postprocessing. **A** Input image. **B** Result of segmentation. The spot surrounded by red circle is the noise point in the segmentation result. **C** Result of postprocessing

There may be some small noise spots in the automatic segmentation results, which will affect the positioning of the landmarks on the ST. Therefore, before automatic measurement, the binary image generated by the segmentation network needs to be further processed to remove the noise spots in the binary images. Because the ST is unique in the cephalometric radiographs and only a few segmentation results have small noise points, the method that only preserves the maximum connected region can be used for postprocessing the segmentation results. The postprocessing operation first uses the “findCountours” function in the OpenCV library to detect the boundaries of all separate objects in the binary images, and then only preserves the single boundary with the maximum number of pixels inside. Finally, after the postprocessing operation, the noise spots in the binary images were removed, and the only white connected region was the corresponding ST. Figure 5 shows an example of postprocessing. Based on these postprocessed binary images, the automatic morphological measurement of ST was further carried out.

### Automatic measurement of linear parameters

According to the labeling rules of manual segmentation of ST, the four landmarks of ST are located on the contour of the segmentation result. The morphological operation in the OpenCV library was used to automatically locate the four landmarks on binary images. The binary images were generated by the automatic segmentation network and post-processed. After locating the four landmarks of the ST, the Length, Diameter and Depth of the ST can be obtained by simple distance calculation.

1. Location of landmarks

ADP and BPF were located by detecting the extremum points of the contour. Firstly, the “findCountours” function was used to detect the contour of the binary image, and the coordinates of all contour points can be obtained. Then the coordinates of the top, bottom, left and right extremum points of the contour can be calculated in the set of contour points. Among the four extremum points, the left-most point is ADP, and the bottom-most point is BPF. The detected ADP and BPF are marked on the image and their corresponding pixel coordinates are returned.

**Fig.6:**
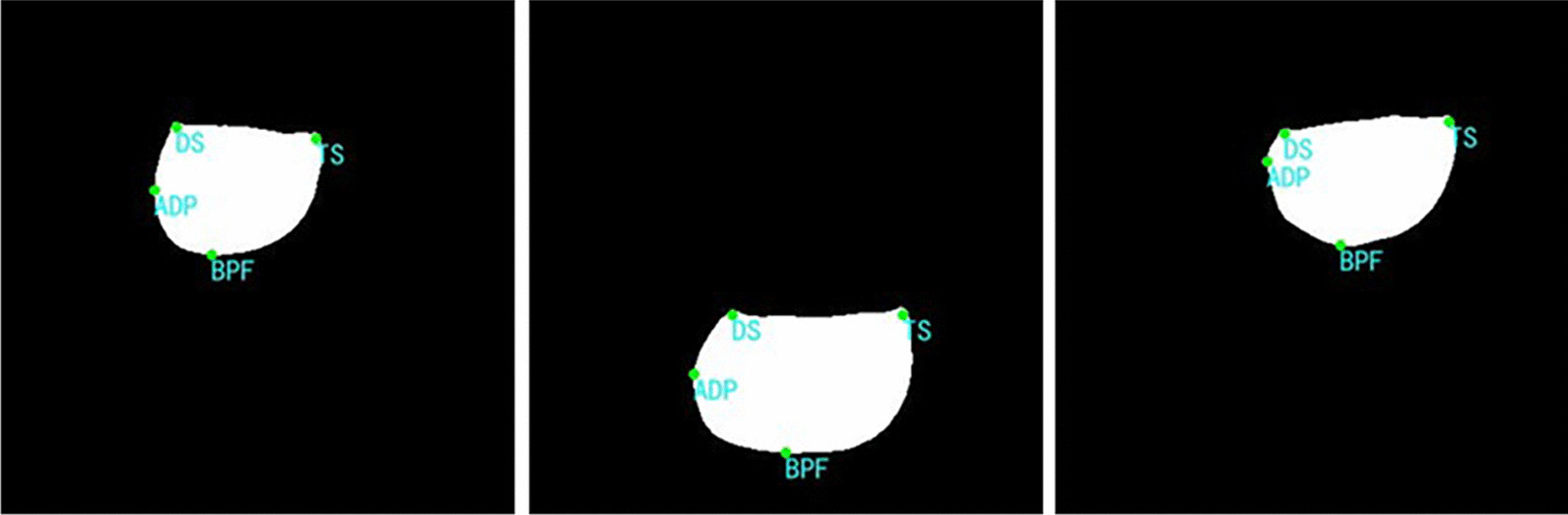
Examples of automatic positioning of landmarks on binary images

TS and DS are located by the Shi-Tomasi corner point detection algorithm. According to the position characteristics of DS and TS in binary images, it can be found that these two points belong to corner points. Shi-Tomasi corner point detection can be realized by calling the “goodFeaturesToTrack” function in the OpenCV library. However, it should be noted that the segmentation results generated by the segmentation network are saved in the form of binary images, in which the edge of the white connected region representing the ST is serrated. In corner point detection, it is easy to identify the serrations on the edge as corner points, resulting in the wrong positioning of DS and TS. Therefore, when locating TS and DS, we first use the Gaussian filtering algorithm to smooth the image and then carry out corner points detection on the smoothed image. In addition, setting the proper parameters of the “goodFeaturesToTrack” function is also critical to locating DS and TS points correctly. Through many experiments, it was found that when maxCorners, qualityLevel, minDistance, and blocksize of the “goodFeaturesToTrack” function are set to 3, 0.01, 40, and 5, TS and DS in almost all images will be correctly detected. In the OpenCV coordinate system, the origin of coordinates 0(0, 0) is in the upper left corner of the image. In this study, the two corner points with the smallest ordinate were screened out from the detected corner points, and then the abscissa of the two points was compared. The point with the larger abscissa is TS, and the point with the smaller abscissa is DS. TS and DS were marked in the image and their corresponding pixel coordinates were returned. The four landmarks of ST automatic positioning on binary images are shown in Fig. 6.

2. Distance calculation

After automatically locating the four landmarks of the ST, we obtained their pixel coordinates, denoted as ${\text{TS}}\left( {x_{1} ,y_{1} } \right)$, ${\text{DS}}\left( {x_{2} ,y_{2} } \right)$, ${\text{ADP}}\left( {x_{3} ,y_{3} } \right)$, and ${\text{BPF}}\left( {x_{4} ,y_{4} } \right)$. Next, the values of the linear parameters of ST can be easily calculated based on the formula for the distance between two points and the formula for the distance from the point to the line. The length of the ST can be obtained by calculating the distance between TS and DS, the diameter of the ST can be obtained by calculating the distance between TS and ADP, and the depth of the ST can be obtained by calculating the vertical distance from BPF to a straight line formed by TS and DS connections. The specific calculation formula is as follows:

2 $$Length = \sqrt {\left( {x_{2} - x_{1} } \right)^{2} + \left( {y_{2} - y_{1} } \right)^{2} } \times R$$

3 $$Diameter = \sqrt {\left( {x_{3} - x_{1} } \right)^{2} + \left( {y_{3} - y_{1} } \right)^{2} } \times R$$

4 $$Depth = \frac{{\left| {\left( {y_{1} - y_{2} } \right)x_{4} + \left( {x_{2} - x_{1} } \right)y_{4} + x_{1} y_{2} - y_{1} x_{2} } \right|}}{{\sqrt {\left( {y_{2} - y_{1} } \right)^{2} + \left( {x_{2} - x_{1} } \right)^{2} } }} \times R$$

The scale (R) was determined by its ratio of actual diameter to pixel diameter. In this study, the value of R is 0.1. The scale was used to convert the pixel distances of ST measurements into the actual distance in millimeters.

### Statistical analyses

The segmentation performance of ST was evaluated by dice coefficient. Intraclass correlation coefficients (ICCs) and Bland–Altman plots were used to evaluate the reliability of the proposed automatic measurement method. The results of manual and automatic measurements for all images in Test1 and Test2 were recorded in Excel. The ICCs of the manual and automatic measurements were calculated using *SPSS* (version 26). ICC is a popular indicator used to assess agreement between quantitative measurements taken from different observers, with a value between 0 and 1 [26]. It was considered moderate agreement if 0.41 < ICC ≤ 0.60, substantial agreement if 0.60 < ICC ≤ 0.80, and excellent agreement if 0.80 < ICC ≤ 1.00 [19]. Bland–Altman analysis is a way to visually demonstrate consistency using graphics [27]. *GraphPad Prism* (version 9.0.0) was used to plot Bland–Altman plots to show the agreement between automatic and manual measurements of ST linear parameters.

## Results

### Performance of automatic segmentation

**Fig.7:**
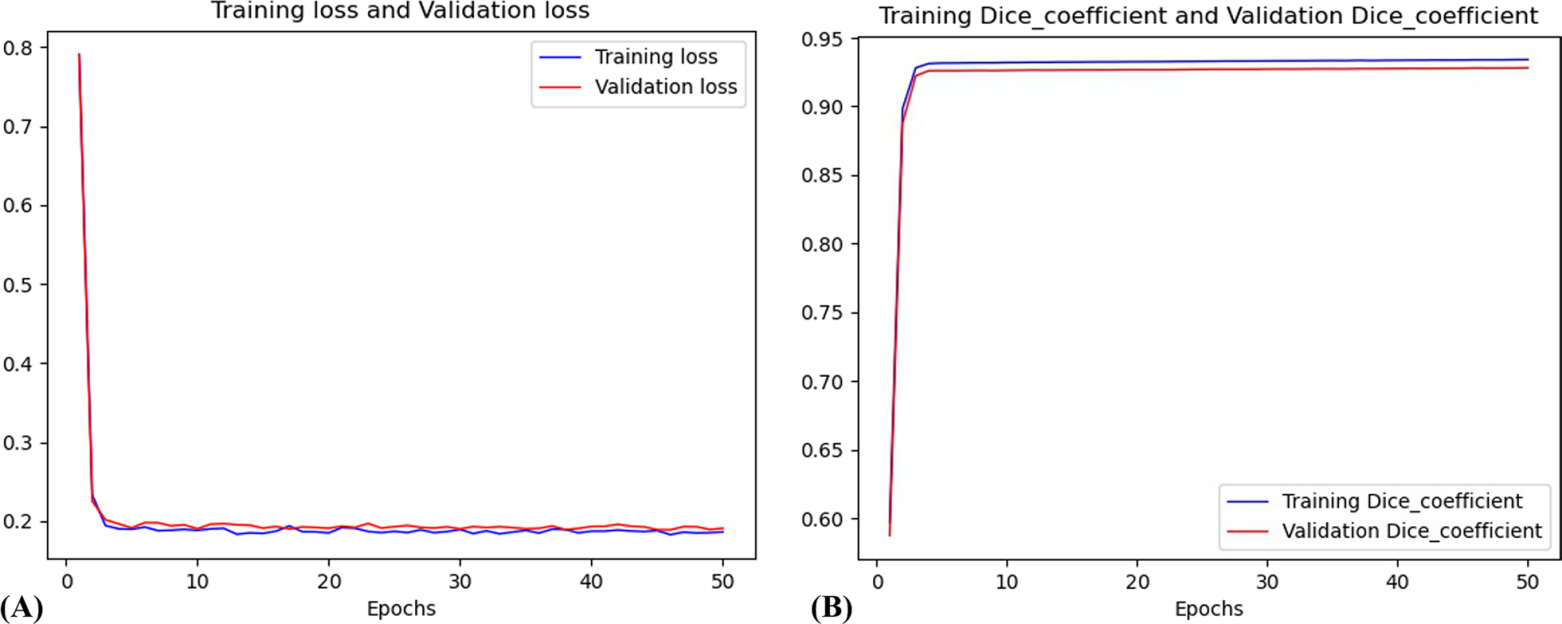
The training graph of the U-net model. **A** Training loss and Validation loss. **B** Training dice coefficient and Validation dice coefficient

In this study, the U-net was trained by using 918 images in the training dataset until there was no more improvement in the dice coefficient. The loss curve and dice coefficient curve during the training of U-net model are shown in Fig. 7. It can be seen from the training graph that in the 1–3 epochs, the convergence rate of loss is fast, and dice coefficient has exceeded 0.9. With more epochs, dice coefficient improved slightly.

**Fig.8:**
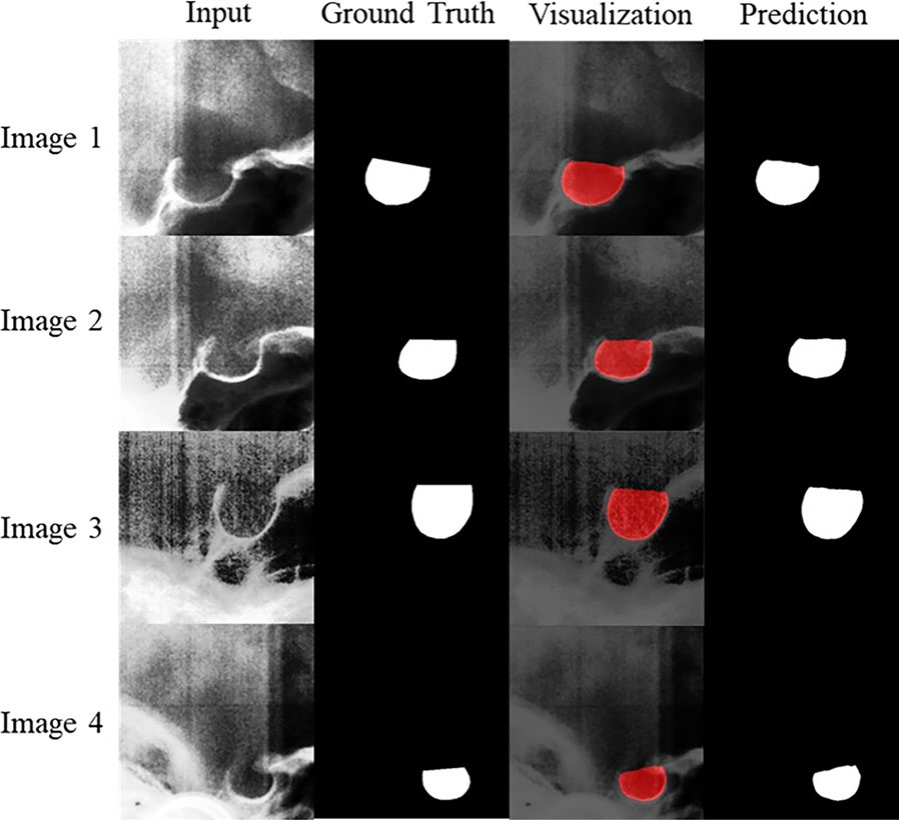
Examples of automatic segmentation results

After the training of 50 epochs, the segmentation network achieved 93.44% dice coefficient on the training dataset and 92.84% dice coefficient on the validation dataset. The models that perform best on the validation dataset were used to segment the images in the test dataset. The segmentation network can complete automatic segmentation of an image in half a second. Figure 8 presents multiple input images from the testing dataset, the ground truth acquired through manual annotation, the visual results of image segmentation with the segmentation network, and the predicted ST segmentation results.

### Reliability of automatic measurement

110 images from Test1 and 50 images from Test2 were measured, the results of manual and automatic measurement of linear parameters of ST were shown in Table 1, and measurement data were expressed as “mean ± standard deviation”.

**Table 1:** Measurement results of linear parameters of ST

Data	Measurement	Length (mm)	Diameter (mm)	Depth (mm)
Test1(110)	Manual	10.52 ± 1.54	12.69 ± 1.22	8.57 ± 1.07
	Automatic	10.45 ± 1.47	12.58 ± 1.21	8.41 ± 1.08
Test2(50)	Manual	8.66 ± 0.1	10.45 ± 0.97	6.83 ± 0.97
	Automatic	8.81 ± 1.12	10.55 ± 0.1	6.71 ± 1.02

**Fig.9:**
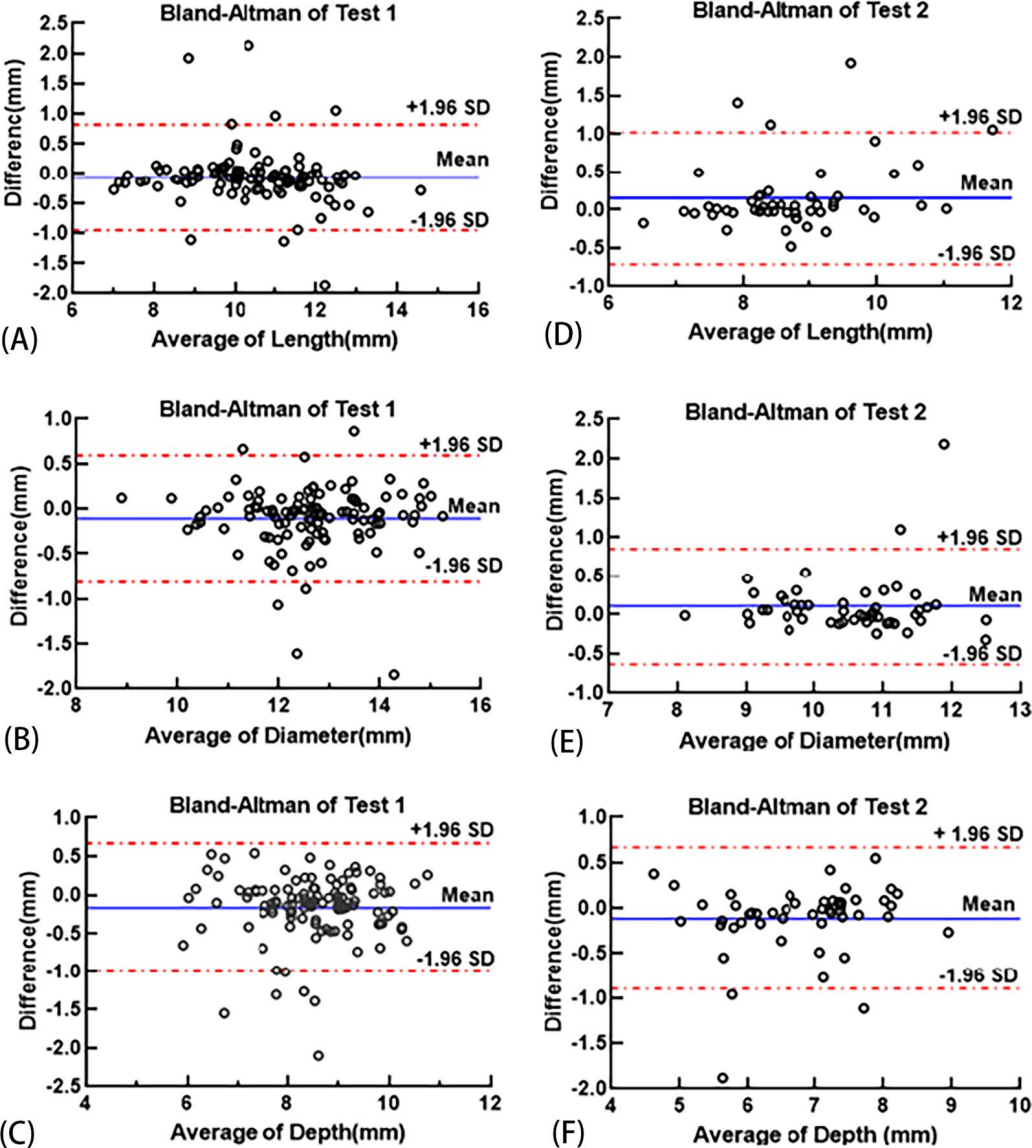
Bland–Altman Plots analyze the agreement between manual and automatic measurements. The three pictures on the left are the results of Test1. **A** Agreement between manual Length and automated Length. **B** Agreement between manual Diameter and automated Diameter. **C** Agreement between manual Depth and automated Depth. The three pictures on the right are the results of Test2. (**D**). Agreement between manual Length and automated Length. **E** Agreement between manual Diameter and automated Diameter. **F** Agreement between manual Depth and automated Depth

According to the statistical analysis, the results obtained manually by the *Digimizer* and the results obtained automatically by the proposed method are in excellent agreement. For all images in Test1, the ICCs of Length obtained by manual and automatic measurements were 0.954, the ICCs of Diameter obtained by manual and automatic measurements were 0.953, and the ICCs of Depth obtained by manual and automatic measurements were 0.912. Similarly, excellent agreement was achieved in Test2, with ICCs of 0.906 for Length, 0.921 for Diameter, and 0.915 for Depth. Bland–Altman analysis also showed excellent agreement, with most of the measured differences within the 95% limits of agreement, as shown in Fig. 9. For Test1, the 95% limits of agreement (LoA) were − 0.955 to 0.819 mm for Length, − 0.808 to 0.593 mm for Diameter and − 0.998 to 0.668 mm for Depth. For Test2, the 95% limits of agreement (LoA) were − 0.717 to 1.017 mm for Length, − 0.638 to 0.849 mm for Diameter, and − 0.899 to 0.666 mm for Depth.

## Discussion

In this preliminary study, we proposed and evaluated a novel method for automatic segmentation and measurement of ST based on the deep learning algorithm. Firstly, U-net was trained with labeled images in the training dataset to generate a segmentation network that could perform automatic segmentation of ST efficiently. The trained segmentation network automatically segmented the images in the test datasets, and the segmentation results were saved in the form of binary images. Then, the function in the OpenCV library was called to automatically locate the four landmarks of the ST on the binary images and return their coordinates. Finally, the linear parameters of the ST were obtained by calculating the distance between two points and the distance from a point to the line. The proposed method can simultaneously obtain the length, diameter, and depth of ST in one second, and the results obtained automatically are in excellent agreement with those obtained manually using *Digimizer*. The good performance on Test2 shows that the proposed methodology presented generalization ability. This automatic measurement method is efficient and reliable in measuring ST on cephalometric radiographs and it is a powerful tool to promote the study of ST morphology. In addition, this study firstly segmented the image, then located the landmarks based on the segmentation results, and finally realized automatic measurement through distance calculation, which may provide new ideas for automatic measurement of other medical images.

There are a few studies on automatic segmentation and measurement of ST based on deep learning. Shakya et al. proposed a novel technique using convolutional neural network (CNN) architectures for automatic segmentation of sella turcica (ST) on cephalometric radiographic image dataset [28]. Their study compared training and prediction results of the selected models: VGG19, ResNet34, InceptionV3, and ResNext50. The dice coefficients are 0.7794, 0.7487, 0.4714, 0.4363, respectively. The dice coefficient obtained by our proposed method was 0.9284. Although the dataset we use is different from Shakya’s, our method gets higher dice coefficients than theirs. In addition, our study is the first to achieve automatic measurement of the length, depth, and diameter of the ST, and obtain reliable measurement results.

ST plays an important suggestive role in helping clinicians predict patients' craniofacial development, assess craniofacial morphological characteristics, and find abnormal or pathological changes in the pituitary gland. Therefore, it is of great significance to study the morphological changes and growth rules of ST. The evaluation of ST morphology is inseparable from the measurement of linear parameters of ST. With the development of science and technology, the tools for measuring ST are improving. In the early years, researchers used tracing paper to outline the ST and then used digital calipers to measure the linear parameters of the ST [29, 30]. In recent years, some digital measurement software greatly facilitates the researchers to measure ST [31, 32]. However, manual measurement, whether by tracing paper or digital software, is time-consuming and laborious, and is prone to inter-observer and intra-observer deviation. Deep learning algorithm has made many achievements in the field of medical image processing in recent years, which provides the possibility for automatic measurement of ST. We developed a reliable automatic measurement tool based on deep learning for automatic segmentation and measurement of ST. According to data on the size of ST reported in the literature. It typically ranges from 4 to 12 mm for the vertical and from 5 to 16 mm for the anteroposterior dimension [33]. The measurement results of this study are consistent with the data of previous studies, which demonstrates the accuracy of our measurement results.

There are some limitations in our study. Firstly, the number of images used in our experiment is small. Although a lightweight dataset may be adequate to train the U-net to segment ST with satisfactory segmentation performance, for deep learning algorithms, the addition of training samples can theoretically improve the segmentation performance of the model. Secondly, because most of the studies on ST morphology are carried out on two-dimensional images, and the quantitative measurement standards on two-dimensional images are relatively uniform, this study has taken two-dimensional images as the research object. But with the clinical application of CBCT technology, some researchers have begun to pay attention to the ST on three-dimensional images [1, 32]. The morphological parameters reflected in the 3D images may be more realistic. Thirdly, although the linear parameters of the ST were taken into consideration in our study, some non-quantitative parameters are needed to be integrated into the automatic system in the further studies, such as the bridging and shape of the ST. Fourthly, the preprocessing operation of ROI extraction needs to be completed based on coordinate parameters. Therefore, when processing different datasets, coordinate parameters may need to be changed according to the characteristics of the images. Automatic ROI extraction based on deep learning network may be more ideal. In future work, we will try to realize automatic ROI extraction without setting coordinate parameters.

This preliminary study was explored the possibility of realizing automatic segmentation of ST based on deep learning, and the classical U-net was selected as the segmentation network. Although the method proposed by us has effectively completed the automatic segmentation of the ST, we believe that this study can be further improved in the future work. If more images are used to train the deep learning network, or the algorithm is improved, such as adding the attention mechanism or residual structure, better experimental results may be obtained. We learned that there are many improved algorithms of U-net, such as nnU-Net, U-Net +  + and UNET3 + , and so on [24]. These improved algorithms have better segmentation performance on some datasets than U-net. In future work, we will try to improve the algorithm to obtain better results. Besides, the method of automatic measurement of ST morphology needs to be further explored and extended. More specifically, although the proposed method enables accurate quantitative measurements, the measurement procedure requires a workstation to run and cannot be directly applied to the clinic. Our team will strive to build more complete automated measurement programs to be easy to embed into the digital image analysis software. At the same time, we consider integrating more parameters into automated analysis systems, which may require more deep learning algorithms and computer vision-related technologies.

## Conclusions

This study developed and validated a method based on deep learning algorithm and OpenCV library for automatic segmentation and measurement of ST. The proposed method can predict the length, diameter, and depth of ST quickly, and the automatic measurement results are in excellent agreement with the manual measurement results. The efficient and reliable automatic measurement method can facilitate the study of ST morphology.

## Data Availability

The datasets used and analysed during the current study are available from the corresponding author on reasonable request.

## Abbreviations

ST: Sella turcica
PACS: Picture archiving and communication system
ICCs: Intraclass correlation coefficients
TS: Tuberculum sella
DS: Dorsum sella
ADP: Anteroposterior diameter
BPF: Base of the pituitary fossa
ROI: Region of interest
LoA: Limits of agreement
